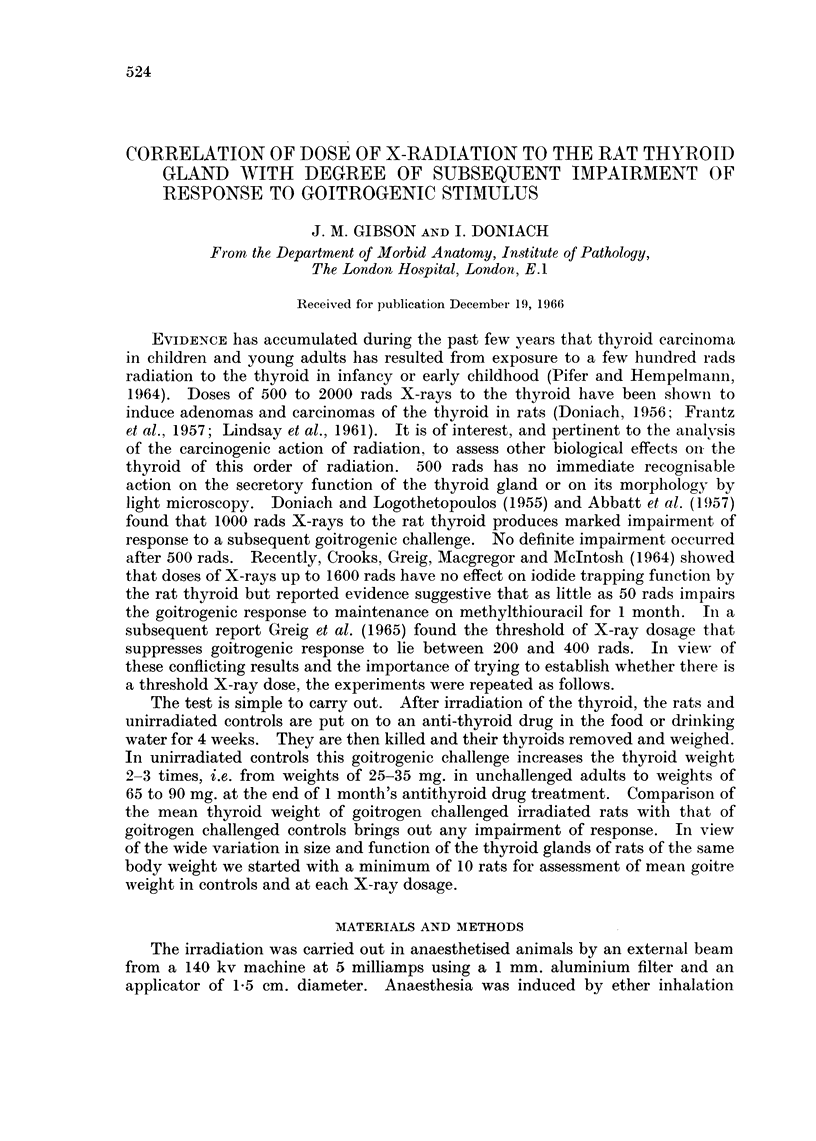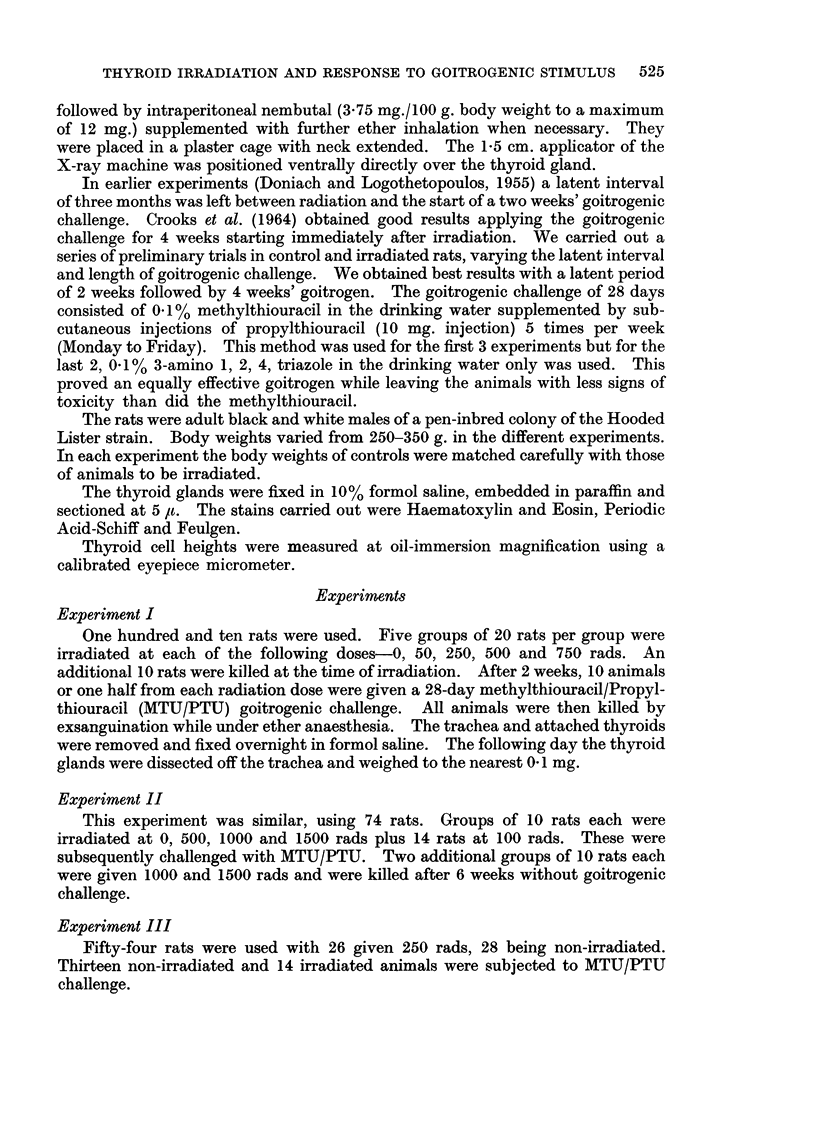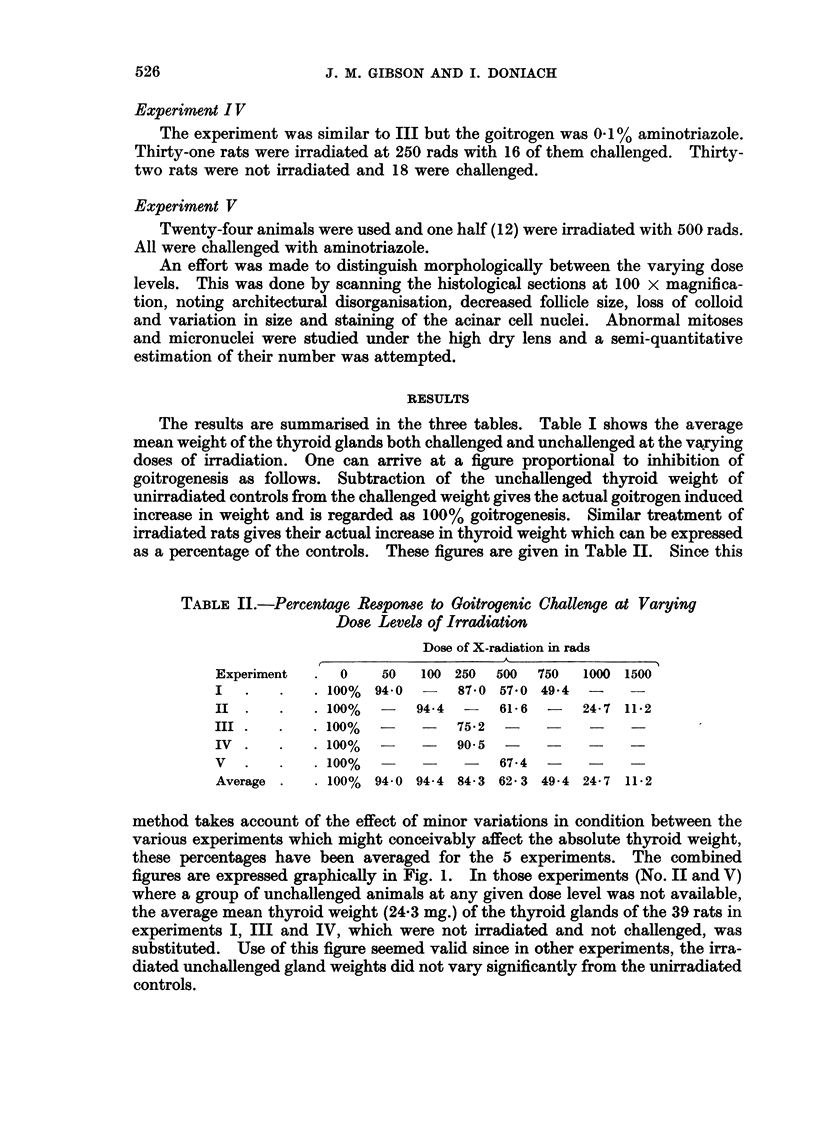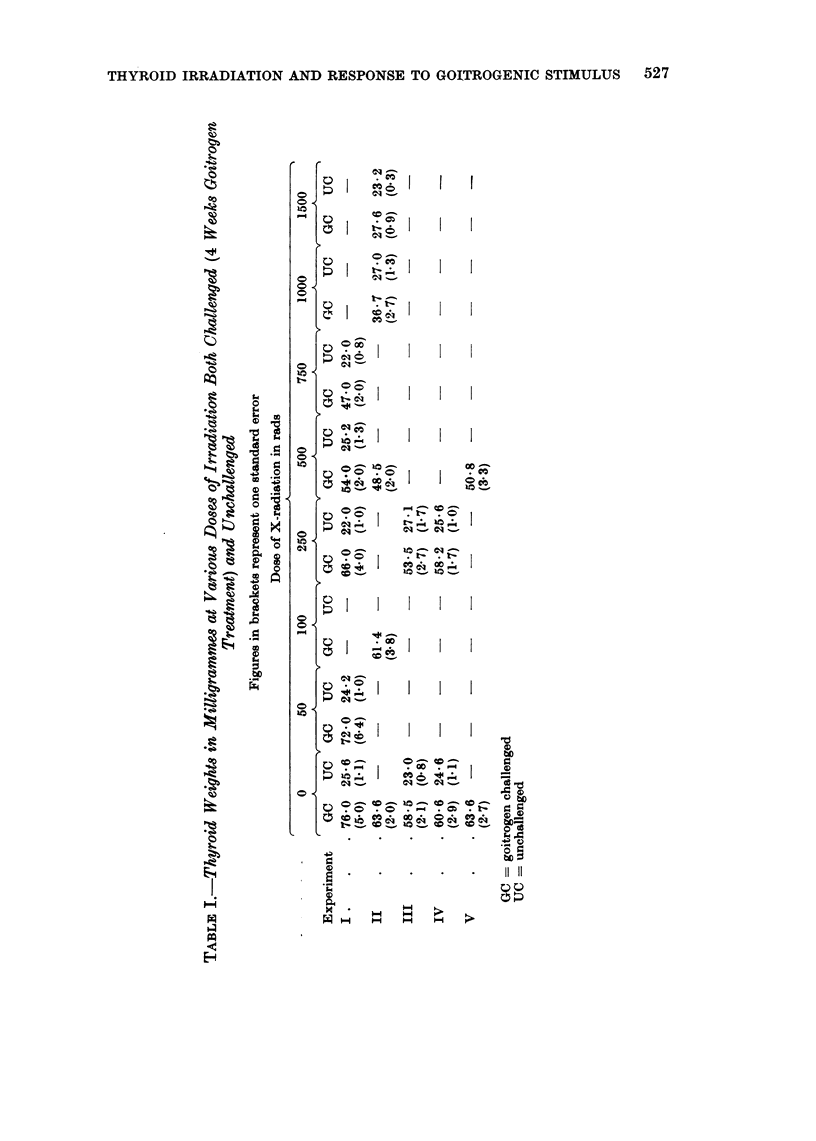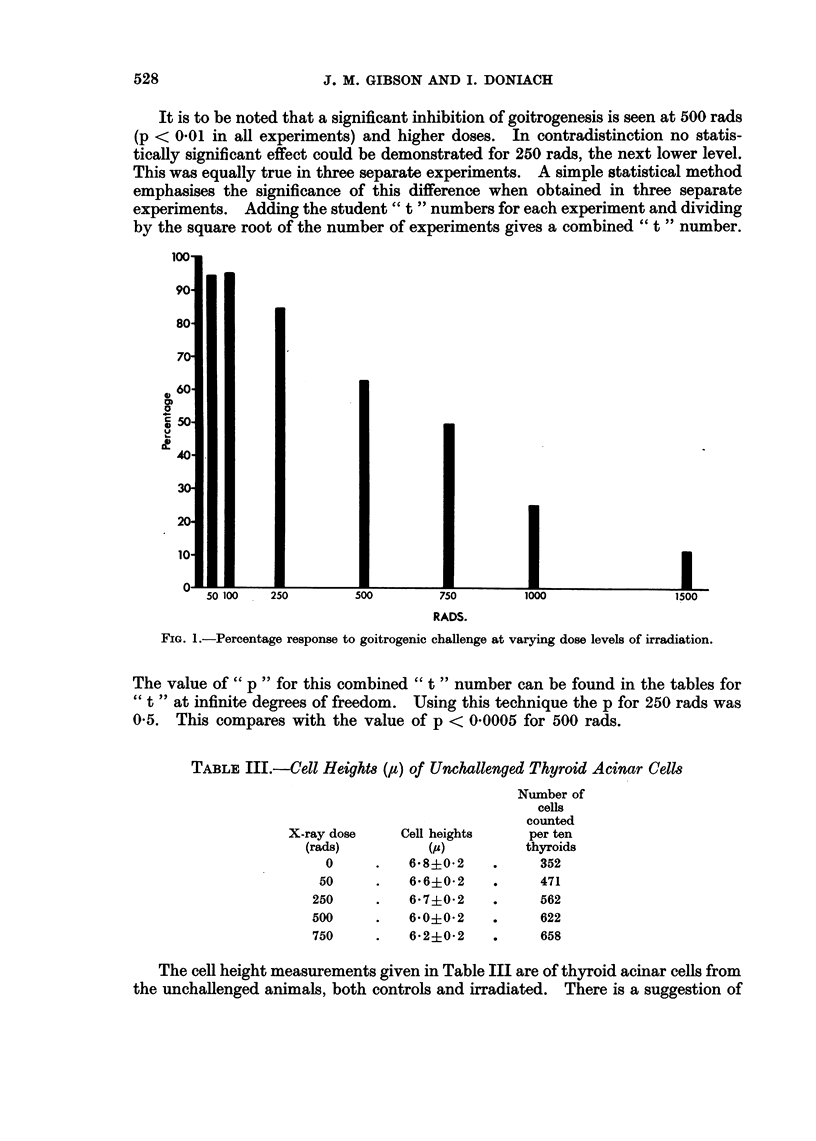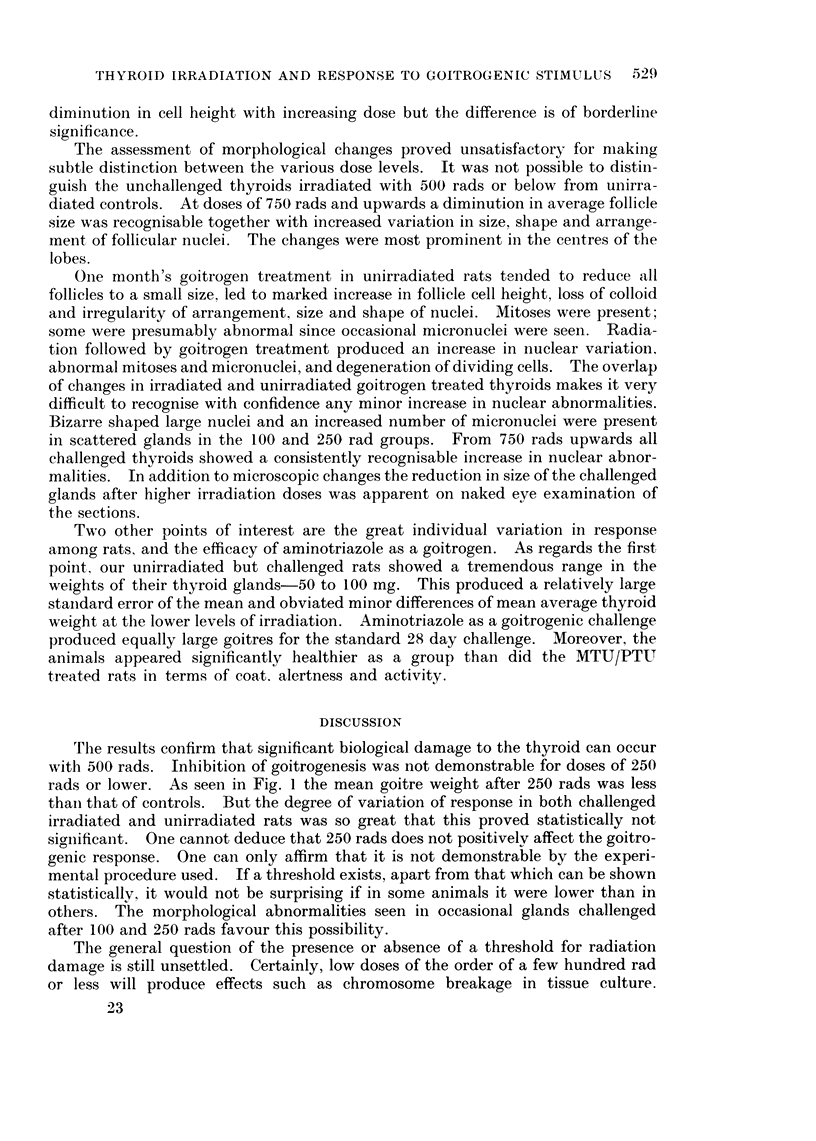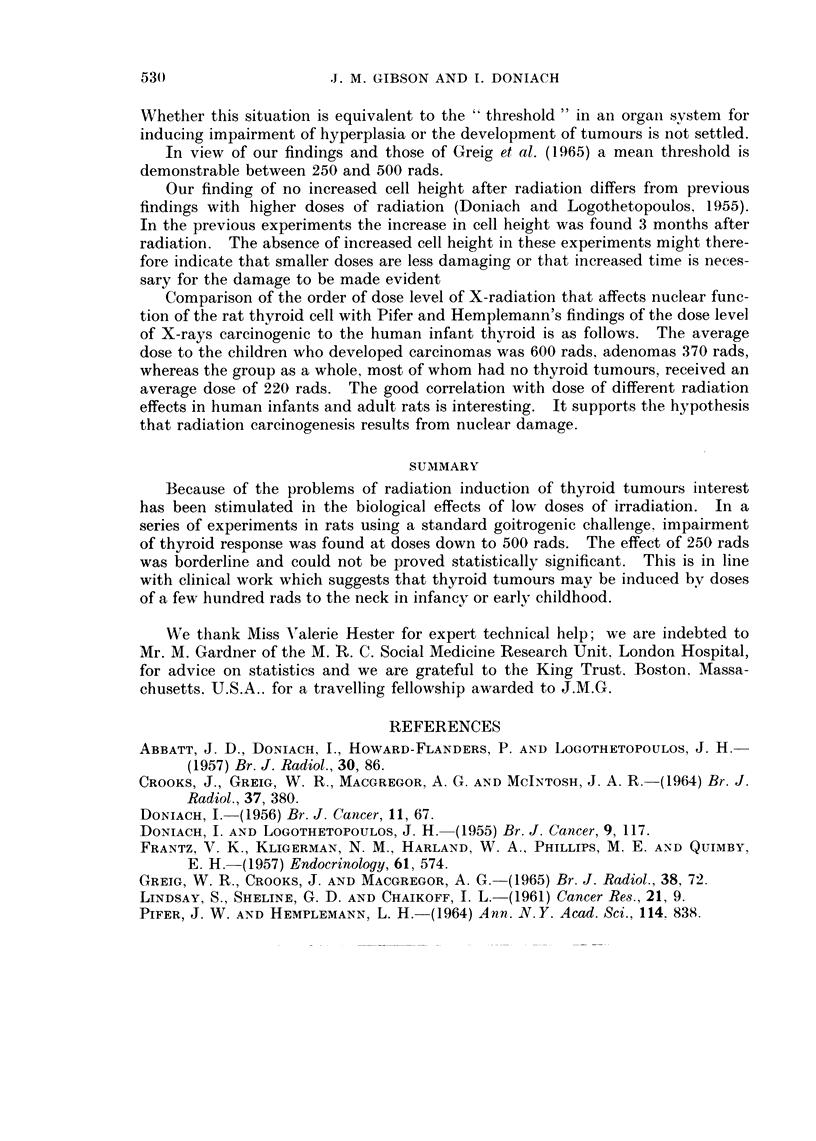# Correlation of dose of x-radiation to the rat thyroid gland with degree of subsequent impairment of response to goitrogenic stimulus.

**DOI:** 10.1038/bjc.1967.62

**Published:** 1967-09

**Authors:** J. M. Gibson, I. Doniach


### CORRELATION OF DOSE OF X-RADIATION TO THE RAT THYROID GLAND WITH DEGREE OF SUBSEQUENT IMPAIRMENT OF RESPONSE TO GOITROGENIC STIMULUS

J. M. GIBSON AND I. DONIACH

*From the Department of Morbid Anatomy, Institute of Pathology, The London Hospital, London, E.1*

Received for publication December 19, 1966

EVIDENCE has accumulated during the past few years that thyroid carcinoma in children and young adults has resulted from exposure to a few hundred rads radiation to the thyroid in infancy or early childhood (Pifer and Hempelmann, 1964). Doses of 500 to 2000 rads X-rays to the thyroid have been shown to induce adenomas and carcinomas of the thyroid in rats (Doniach, 1956; Frantz *et al.*, 1957; Lindsay *et al.*, 1961). It is of interest, and pertinent to the analysis of the carcinogenic action of radiation, to assess other biological effects on the thyroid of this order of radiation. 500 rads has no immediate recognisable action on the secretory function of the thyroid gland or on its morphology by light microscopy. Doniach and Logothetopoulos (1955) and Abbatt *et al.* (1957) found that 1000 rads X-rays to the rat thyroid produces marked impairment of response to a subsequent goitrogenic challenge. No definite impairment occurred after 500 rads. Recently, Crooks, Greig, Macgregor and McIntosh (1964) showed that doses of X-rays up to 1600 rads have no effect on iodide trapping function by the rat thyroid but reported evidence suggestive that as little as 50 rads impairs the goitrogenic response to maintenance on methylthiouracil for 1 month. In a subsequent report Greig *et al.* (1965) found the threshold of X-ray dosage that suppresses goitrogenic response to lie between 200 and 400 rads. In view of these conflicting results and the importance of trying to establish whether there is a threshold X-ray dose, the experiments were repeated as follows.

The test is simple to carry out. After irradiation of the thyroid, the rats and unirradiated controls are put on to an anti-thyroid drug in the food or drinking water for 4 weeks. They are then killed and their thyroids removed and weighed. In unirradiated controls this goitrogenic challenge increases the thyroid weight 2–3 times, *i.e.* from weights of 25–35 mg. in unchallenged adults to weights of 65 to 90 mg. at the end of 1 month's antithyroid drug treatment. Comparison of the mean thyroid weight of goitrogen challenged irradiated rats with that of goitrogen challenged controls brings out any impairment of response. In view of the wide variation in size and function of the thyroid glands of rats of the same body weight we started with a minimum of 10 rats for assessment of mean goitre weight in controls and at each X-ray dosage.

### MATERIALS AND METHODS

The irradiation was carried out in anaesthetised animals by an external beam from a 140 kv machine at 5 milliamps using a 1 mm. aluminium filter and an applicator of 1·5 cm. diameter. Anaesthesia was induced by ether inhalation followed by intraperitoneal nembutal (3·75 mg./100 g. body weight to a maximum of 12 mg.) supplemented with further ether inhalation when necessary. They were placed in a plaster cage with neck extended. The 1·5 cm. applicator of the X-ray machine was positioned ventrally directly over the thyroid gland.

In earlier experiments (Doniach and Logothetopoulos, 1955) a latent interval of three months was left between radiation and the start of a two weeks' goitrogenic challenge. Crooks *et al.* (1964) obtained good results applying the goitrogenic challenge for 4 weeks starting immediately after irradiation. We carried out a series of preliminary trials in control and irradiated rats, varying the latent interval and length of goitrogenic challenge. We obtained best results with a latent period of 2 weeks followed by 4 weeks' goitrogen. The goitrogenic challenge of 28 days consisted of 0·1% methylthiouracil in the drinking water supplemented by subcutaneous injections of propylthiouracil (10 mg. injection) 5 times per week (Monday to Friday). This method was used for the first 3 experiments but for the last 2, 0·1% 3-amino 1, 2, 4, triazole in the drinking water only was used. This proved an equally effective goitrogen while leaving the animals with less signs of toxicity than did the methylthiouracil.

The rats were adult black and white males of a pen-inbred colony of the Hooded Lister strain. Body weights varied from 250–350 g. in the different experiments. In each experiment the body weights of controls were matched carefully with those of animals to be irradiated.

The thyroid glands were fixed in 10% formol saline, embedded in paraffin and sectioned at 5 $\mu$. The stains carried out were Haematoxylin and Eosin, Periodic Acid-Schiff and Feulgen.

Thyroid cell heights were measured at oil-immersion magnification using a calibrated eyepiece micrometer.

### *Experiments*

### *Experiment I*

One hundred and ten rats were used. Five groups of 20 rats per group were irradiated at each of the following doses—0, 50, 250, 500 and 750 rads. An additional 10 rats were killed at the time of irradiation. After 2 weeks, 10 animals or one half from each radiation dose were given a 28-day methylthiouracil/Propylthiouracil (MTU/PTU) goitrogenic challenge. All animals were then killed by exsanguination while under ether anaesthesia. The trachea and attached thyroids were removed and fixed overnight in formol saline. The following day the thyroid glands were dissected off the trachea and weighed to the nearest 0·1 mg.

### *Experiment II*

This experiment was similar, using 74 rats. Groups of 10 rats each were irradiated at 0, 500, 1000 and 1500 rads plus 14 rats at 100 rads. These were subsequently challenged with MTU/PTU. Two additional groups of 10 rats each were given 1000 and 1500 rads and were killed after 6 weeks without goitrogenic challenge.

### *Experiment III*

Fifty-four rats were used with 26 given 250 rads, 28 being non-irradiated. Thirteen non-irradiated and 14 irradiated animals were subjected to MTU/PTU challenge.

*Experiment IV*

The experiment was similar to III but the goitrogen was 0·1% aminotriazole. Thirty-one rats were irradiated at 250 rads with 16 of them challenged. Thirty-two rats were not irradiated and 18 were challenged.

*Experiment V*

Twenty-four animals were used and one half (12) were irradiated with 500 rads. All were challenged with aminotriazole.

An effort was made to distinguish morphologically between the varying dose levels. This was done by scanning the histological sections at 100 × magnification, noting architectural disorganisation, decreased follicle size, loss of colloid and variation in size and staining of the acinar cell nuclei. Abnormal mitoses and micronuclei were studied under the high dry lens and a semi-quantitative estimation of their number was attempted.

### RESULTS

The results are summarised in the three tables. Table I shows the average mean weight of the thyroid glands both challenged and unchallenged at the varying doses of irradiation. One can arrive at a figure proportional to inhibition of goitrogenesis as follows. Subtraction of the unchallenged thyroid weight of unirradiated controls from the challenged weight gives the actual goitrogen induced increase in weight and is regarded as 100% goitrogenesis. Similar treatment of irradiated rats gives their actual increase in thyroid weight which can be expressed as a percentage of the controls. These figures are given in Table II. Since this

TABLE II.—*Percentage Response to Goitrogenic Challenge at Varying Dose Levels of Irradiation*

| | Dose of X-radiation in rads | | | | | | | |
|---|---|---|---|---|---|---|---|---|
| Experiment | 0 | 50 | 100 | 250 | 500 | 750 | 1000 | 1500 |
| I | 100% | 94·0 | — | 87·0 | 57·0 | 49·4 | — | — |
| II | 100% | — | 94·4 | — | 61·6 | — | 24·7 | 11·2 |
| III | 100% | — | — | 75·2 | — | — | — | — |
| IV | 100% | — | — | 90·5 | — | — | — | — |
| V | 100% | — | — | — | 67·4 | — | — | — |
| Average | 100% | 94·0 | 94·4 | 84·3 | 62·3 | 49·4 | 24·7 | 11·2 |

method takes account of the effect of minor variations in condition between the various experiments which might conceivably affect the absolute thyroid weight, these percentages have been averaged for the 5 experiments. The combined figures are expressed graphically in Fig. 1. In those experiments (No. II and V) where a group of unchallenged animals at any given dose level was not available, the average mean thyroid weight (24·3 mg.) of the thyroid glands of the 39 rats in experiments I, III and IV, which were not irradiated and not challenged, was substituted. Use of this figure seemed valid since in other experiments, the irradiated unchallenged gland weights did not vary significantly from the unirradiated controls.

TABLE I.—*Thyroid Weights in Milligrammes at Various Doses of Irradiation Both Challenged (4 Weeks Goitrogen Treatment) and Unchallenged*

Figures in brackets represent one standard error

Dose of X-radiation in rads

| | 0 | | 50 | | 100 | | 250 | | 500 | | 750 | | 1000 | | 1500 | |
|---|---|---|---|---|---|---|---|---|---|---|---|---|---|---|---|---|
| Experiment | GC | UC | GC | UC | GC | UC | GC | UC | GC | UC | GC | UC | GC | UC | GC | UC |
| I . . | 76·0 (5·0) | 25·6 (1·1) | 72·0 (6·4) | 24·2 (1·0) | — | — | 66·0 (4·0) | 22·0 (1·0) | 54·0 (2·0) | 25·2 (1·3) | 47·0 (2·0) | 22·0 (0·8) | — | — | — | — |
| II . . | 63·6 (2·0) | — | — | — | 61·4 (3·8) | — | — | — | 48·5 (2·0) | — | — | — | 36·7 (2·7) | 27·0 (1·3) | 27·6 (0·9) | 23·2 (0·3) |
| III . . | 58·5 (2·1) | 23·0 (0·8) | — | — | — | — | 53·5 (2·7) | 27·1 (1·7) | — | — | — | — | — | — | — | — |
| IV . . | 60·6 (2·9) | 24·6 (1·1) | — | — | — | — | 58·2 (1·7) | 25·6 (1·0) | — | — | — | — | — | — | — | — |
| V . . | 63·6 (2·7) | — | — | — | — | — | — | — | 50·8 (3·3) | — | — | — | — | — | — | — |

GC = goitrogen challenged

UC = unchallenged

It is to be noted that a significant inhibition of goitrogenesis is seen at 500 rads ($p < 0{\cdot}01$ in all experiments) and higher doses. In contradistinction no statistically significant effect could be demonstrated for 250 rads, the next lower level. This was equally true in three separate experiments. A simple statistical method emphasises the significance of this difference when obtained in three separate experiments. Adding the student " t " numbers for each experiment and dividing by the square root of the number of experiments gives a combined " t " number.

FIG. 1.—Percentage response to goitrogenic challenge at varying dose levels of irradiation.

The value of " p " for this combined " t " number can be found in the tables for " t " at infinite degrees of freedom. Using this technique the p for 250 rads was 0·5. This compares with the value of $p < 0{\cdot}0005$ for 500 rads.

TABLE III.—*Cell Heights (μ) of Unchallenged Thyroid Acinar Cells*

| X-ray dose (rads) | Cell heights (μ) | Number of cells counted per ten thyroids |
|---|---|---|
| 0 | 6·8±0·2 | 352 |
| 50 | 6·6±0·2 | 471 |
| 250 | 6·7±0·2 | 562 |
| 500 | 6·0±0·2 | 622 |
| 750 | 6·2±0·2 | 658 |

The cell height measurements given in Table III are of thyroid acinar cells from the unchallenged animals, both controls and irradiated. There is a suggestion of diminution in cell height with increasing dose but the difference is of borderline significance.

The assessment of morphological changes proved unsatisfactory for making subtle distinction between the various dose levels. It was not possible to distinguish the unchallenged thyroids irradiated with 500 rads or below from unirradiated controls. At doses of 750 rads and upwards a diminution in average follicle size was recognisable together with increased variation in size, shape and arrangement of follicular nuclei. The changes were most prominent in the centres of the lobes.

One month's goitrogen treatment in unirradiated rats tended to reduce all follicles to a small size, led to marked increase in follicle cell height, loss of colloid and irregularity of arrangement, size and shape of nuclei. Mitoses were present; some were presumably abnormal since occasional micronuclei were seen. Radiation followed by goitrogen treatment produced an increase in nuclear variation, abnormal mitoses and micronuclei, and degeneration of dividing cells. The overlap of changes in irradiated and unirradiated goitrogen treated thyroids makes it very difficult to recognise with confidence any minor increase in nuclear abnormalities. Bizarre shaped large nuclei and an increased number of micronuclei were present in scattered glands in the 100 and 250 rad groups. From 750 rads upwards all challenged thyroids showed a consistently recognisable increase in nuclear abnormalities. In addition to microscopic changes the reduction in size of the challenged glands after higher irradiation doses was apparent on naked eye examination of the sections.

Two other points of interest are the great individual variation in response among rats, and the efficacy of aminotriazole as a goitrogen. As regards the first point, our unirradiated but challenged rats showed a tremendous range in the weights of their thyroid glands—50 to 100 mg. This produced a relatively large standard error of the mean and obviated minor differences of mean average thyroid weight at the lower levels of irradiation. Aminotriazole as a goitrogenic challenge produced equally large goitres for the standard 28 day challenge. Moreover, the animals appeared significantly healthier as a group than did the MTU/PTU treated rats in terms of coat, alertness and activity.

### DISCUSSION

The results confirm that significant biological damage to the thyroid can occur with 500 rads. Inhibition of goitrogenesis was not demonstrable for doses of 250 rads or lower. As seen in Fig. 1 the mean goitre weight after 250 rads was less than that of controls. But the degree of variation of response in both challenged irradiated and unirradiated rats was so great that this proved statistically not significant. One cannot deduce that 250 rads does not positively affect the goitrogenic response. One can only affirm that it is not demonstrable by the experimental procedure used. If a threshold exists, apart from that which can be shown statistically, it would not be surprising if in some animals it were lower than in others. The morphological abnormalities seen in occasional glands challenged after 100 and 250 rads favour this possibility.

The general question of the presence or absence of a threshold for radiation damage is still unsettled. Certainly, low doses of the order of a few hundred rad or less will produce effects such as chromosome breakage in tissue culture.

Whether this situation is equivalent to the " threshold " in an organ system for inducing impairment of hyperplasia or the development of tumours is not settled.

In view of our findings and those of Greig *et al.* (1965) a mean threshold is demonstrable between 250 and 500 rads.

Our finding of no increased cell height after radiation differs from previous findings with higher doses of radiation (Doniach and Logothetopoulos, 1955). In the previous experiments the increase in cell height was found 3 months after radiation. The absence of increased cell height in these experiments might therefore indicate that smaller doses are less damaging or that increased time is necessary for the damage to be made evident

Comparison of the order of dose level of X-radiation that affects nuclear function of the rat thyroid cell with Pifer and Hemplemann's findings of the dose level of X-rays carcinogenic to the human infant thyroid is as follows. The average dose to the children who developed carcinomas was 600 rads, adenomas 370 rads, whereas the group as a whole, most of whom had no thyroid tumours, received an average dose of 220 rads. The good correlation with dose of different radiation effects in human infants and adult rats is interesting. It supports the hypothesis that radiation carcinogenesis results from nuclear damage.

### SUMMARY

Because of the problems of radiation induction of thyroid tumours interest has been stimulated in the biological effects of low doses of irradiation. In a series of experiments in rats using a standard goitrogenic challenge, impairment of thyroid response was found at doses down to 500 rads. The effect of 250 rads was borderline and could not be proved statistically significant. This is in line with clinical work which suggests that thyroid tumours may be induced by doses of a few hundred rads to the neck in infancy or early childhood.

We thank Miss Valerie Hester for expert technical help; we are indebted to Mr. M. Gardner of the M. R. C. Social Medicine Research Unit, London Hospital, for advice on statistics and we are grateful to the King Trust, Boston, Massachusetts, U.S.A., for a travelling fellowship awarded to J.M.G.

### REFERENCES

Abbatt, J. D., Doniach, I., Howard-Flanders, P. and Logothetopoulos, J. H.—(1957) *Br. J. Radiol.*, **30**, 86.

Crooks, J., Greig, W. R., Macgregor, A. G. and McIntosh, J. A. R.—(1964) *Br. J. Radiol.*, **37**, 380.

Doniach, I.—(1956) *Br. J. Cancer*, **11**, 67.

Doniach, I. and Logothetopoulos, J. H.—(1955) *Br. J. Cancer*, **9**, 117.

Frantz, V. K., Kligerman, N. M., Harland, W. A., Phillips, M. E. and Quimby, E. H.—(1957) *Endocrinology*, **61**, 574.

Greig, W. R., Crooks, J. and Macgregor, A. G.—(1965) *Br. J. Radiol.*, **38**, 72.

Lindsay, S., Sheline, G. D. and Chaikoff, I. L.—(1961) *Cancer Res.*, **21**, 9.

Pifer, J. W. and Hemplemann, L. H.—(1964) *Ann. N.Y. Acad. Sci.*, **114**, 838.